# Report of the Committee on Necrology of the National Association of Dental Faculties

**Published:** 1897-10

**Authors:** 


### Report of the Committee on Necrology of the National Association of Dental Faculties.

PROF. FRANK ABBOT.

Whereas death has removed from our ranks, Prof. Frank Abbot, of New York, and whereas, on account of his social qualities, his genial companionship, and his ability as a practitioner and teacher of dentistry, we realize the great loss to the profession in his death. Therefore, be it resolved that we tender to his family the sincere sympathy of this Association, and request that these resolutions be spread upon the minutes of the Association. And be it further resolved that a copy of these resolutions be sent to the several dental journals of this country for publication.

DR. FRANCIS PEABODY.

Whereas death has taken from among us, Dr. Francis Peabody, of Louisville, Ky., and whereas, we feel that in his death the profession has sustained the losss of an able practitioner and teacher, therefore be it resolved that we tender to his bereaved family our heartfelt sympathy, and that we cause the resolutions to be entered upon the minutes of this Association, and be it further resolved that a copy of these resolutions be sent to the dental journals for publication.

---

According to the monthly report of the Health Office, the annual mortality-rate per thousand for this city during the month of May, 1897, was 12.11, the population of the city being estimated at 405,000, which is lower than justified by census returns of 1890 and increase from extending the city limits. However, 12.11 per thousand approached very near to the normal of the perfect city Utopia.—*Cincinnati Lancet Clinic.*

The reports from the Health Office at Cincinnati for the last few weeks has shown a death-rate per annum of 10.46 to 12.50 per 1,000. We have nowhere seen in any city with anything like such a population as Cincinnati, so gratifying health condition. The rate per 1,000 usually in our larger cities is from 17 to 22.

